# Oral health and its association with dysphagia severity and nutritional vulnerability after stroke: a structural equation modeling study

**DOI:** 10.1007/s00784-026-06951-3

**Published:** 2026-05-28

**Authors:** Mohit Kothari, Silas Alves-Costa, Susilena Arouche Costa, Abhishek Kumar, Gustavo G. Nascimento, Jørgen Feldbaek Nielsen, Peter Svensson, Simple F. Kothari

**Affiliations:** 1 Hammel Neurorehabilitation Centre and University Research Clinic, Department of Clinical Medicine, Aarhus University, Hammel, Denmark; 2 Department of Clinical and Preventive Odontology, Faculty of Health Sciences, University of Southern Denmark, Odense, Denmark; 3 Dentistry Graduate Program, Federal University of Maranhão, São Luís, MA Brazil; 4 Dentistry Graduate Program, CEUMA University, São Luís, MA Brazil; 5 Division of Oral Diagnostics and Rehabilitation, Department of Dental Medicine, Karolinska Institute, Huddinge, Sweden; 6 Academic Center for Geriatric Dentistry, Stockholm, Sweden; 7 Department of Conservative Dentistry and Endodontics, Dr. D. Y. Patil Dental College and Hospital, Pimpri, Pune India; 8 School of Dentistry, University of Utah, Salt Lake City, UT USA; 9 Faculty of Dentistry, National University of Singapore, Queenstown, Singapore; 10 Department of Clinical Neurophysiology, Department of Clinical Medicine, Aarhus University Hospital &, Aarhus University, Aarhus, Denmark

**Keywords:** Dysphagia, Malnutrition, Nutritional Risk, Oral Health, Orofacial Function, Structural Equation Modelling, Stroke.

## Abstract

**Background:**

Oral health deterioration is common after neurological injury and may contribute to functional impairment beyond the oral cavity. In stroke rehabilitation, impaired oral conditions coexist with orofacial dysfunction and dysphagia, yet the mechanistic pathways linking oral health to swallowing impairment and downstream nutritional consequences remain poorly defined. This study examined the interrelationships between oral health, orofacial function, dysphagia, and malnutrition using structural equation modelling (SEM).

**Methods:**

Ninety-two stroke survivors admitted to a neurorehabilitation center underwent standardized assessments of oral health, orofacial function, dysphagia severity at admission, and nutritional screening at week 4. Oral health and orofacial function were modelled as latent variables. SEM was used to quantify pathways linking oral health to malnutrition risk, with bivariate comparisons stratified by dysphagia status.

**Results:**

Poor oral health was significantly associated with reduced orofacial function (β = −0.41, *p* < 0.001), which in turn was associated with dysphagia severity (β = −0.51, *p* < 0.001). Dysphagia showed a direct association with malnutrition risk (β = 0.31, *p* = 0.031). While poor oral health exerted a direct effect on malnutrition risk (β = 0.33, *p* = 0.023), the indirect pathway linking poor oral health to malnutrition through orofacial dysfunction and dysphagia was not statistically significant (β = 0.063, *p* = 0.091). The model identified a coherent oral–orofacial–swallowing pathway consistent with nutritional vulnerability after stroke.

**Conclusion:**

These findings position oral health as an important factor associated with swallowing impairment and nutritional vulnerability after stroke within a modeled pathway. Integrating oral and orofacial assessments into post-stroke care may support earlier identification of patients at risk for functional decline and systemic complications.

**Clinical Significance:**

Oral health deterioration was associated with malnutrition risk after stroke, directly and via impaired orofacial function and dysphagia. Integrating oral and orofacial function measures into routine post-stroke assessments may improve early risk stratification and support coordinated dental and rehabilitation care.

**Supplementary Information:**

The online version contains supplementary material available at 10.1007/s00784-026-06951-3.

## Introduction

Swallowing impairment, including dysphagia and malnutrition are highly prevalent and prognostically significant complication in individuals with stroke [1]. Together, these conditions affect up to 60–62% of patients during post-stroke rehabilitation and are consistently linked to poorer functional recovery, increased complication rates, longer hospital stays, and higher mortality [2]. While dysphagia is a well-recognized contributor to malnutrition, poor oral health, and orofacial dysfunction have received comparatively less mechanistic investigation [3]. Importantly, dysphagia may not fully explain the heterogeneity in nutritional decline observed during stroke rehabilitation, as this relationship is multifactorial and influenced by stroke severity, comorbidities, age, and premorbid status [4]. The complexity of post-stroke care, involving multiple systems and disciplines, often leads to oral and orofacial health being deprioritized, despite their potential relevance to oral functional determinants of eating efficiency and swallowing safety, and life-threatening complications such as dysphagia, malnutrition, and pneumonia [5–8].

Previous research has demonstrated that individuals with acquired brain injury (ABI), including stroke, exhibit high levels of dental plaque, gingival inflammation, and tongue coating during hospitalization [7, 9, 10]. From an oral functional perspective, these oral conditions may represent modifiable contributors to impaired bolus preparation, reduced sensory feedback, diminished appetite, and reduced feeding endurance. While these oral health changes may be markers of neglect, they can also contribute directly to neuromuscular and sensory alterations in the oropharyngeal tract, thereby exacerbating dysphagia [11]. Dysphagia affects up to 78% of stroke survivors [12] and is a well-established contributor to inadequate nutritional intake and aspiration risk [13]. Overall, these observations are consistent with a sequential pathway in which oral health deterioration may impair orofacial function, contribute to dysphagia, and ultimately be associated with increased malnutrition risk.

**Fig. 1:**
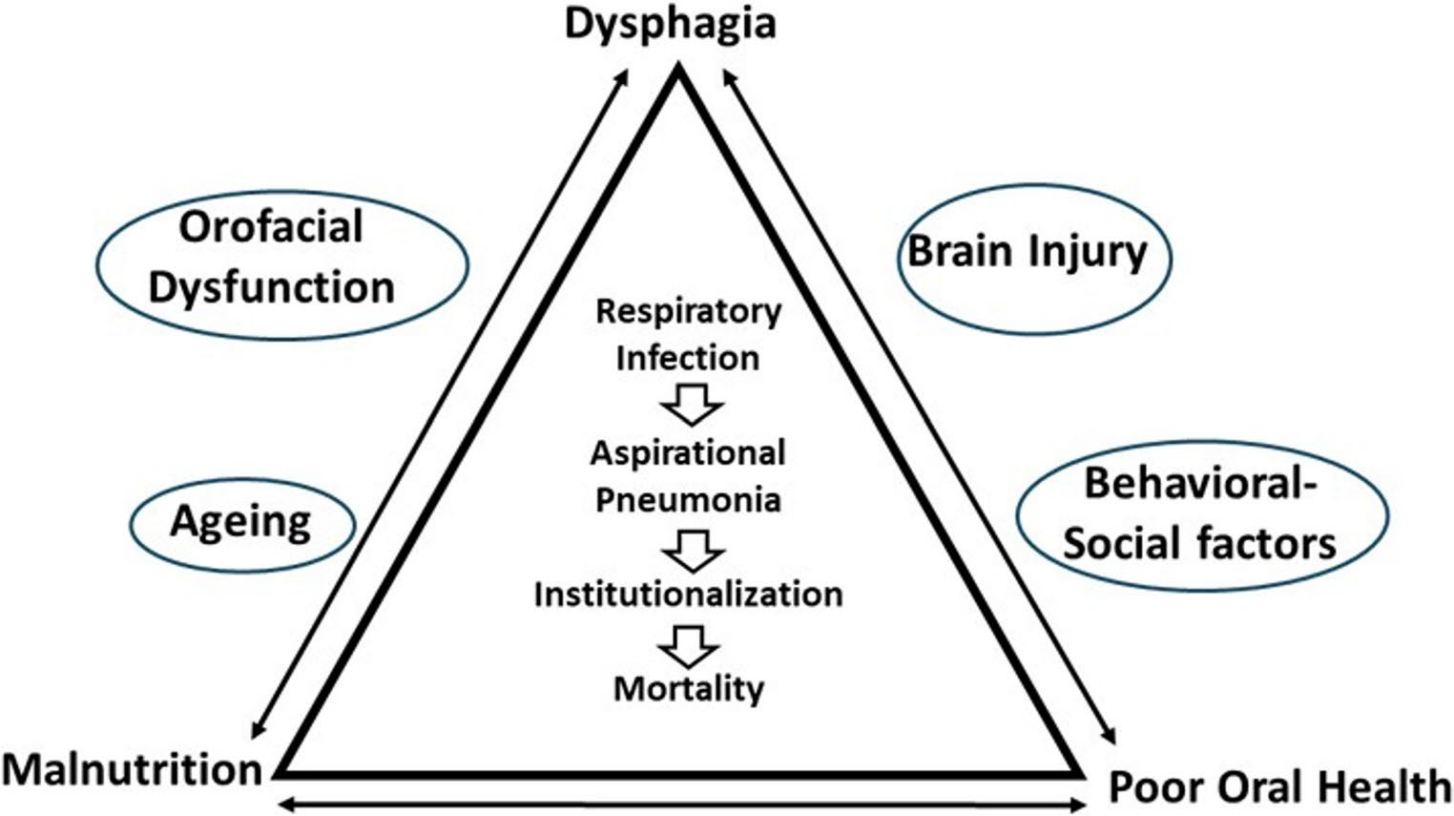
The triangular interplay among dysphagia, poor oral health, and malnutrition in individuals with stroke

A recent study showed a robust association between poor oral health and debilitating conditions like dysphagia and dependency on a feeding tube, which are major risk factors for pneumonia [3]. The study also showed that poor oral health is strongly associated with increasing age within the population and shared common risk factors for ABI, such as smoking, dental visits, and toothbrushing frequency [3], which is also supported by previous research [7, 8]. Beyond biomedical consequences, this triad of dysphagia, poor oral health, and malnutrition may diminish the sensory and social pleasures of eating, with implications for oral health-related quality of life, contributing to social withdrawal, psychological distress, and reduced quality of life (Fig. 1) [3]. From a dental and oral medicine standpoint, integrating oral and orofacial status into functional outcome models is therefore clinically relevant. However, there is a paucity of mechanistic studies in stroke rehabilitation that integrate oral health and orofacial function into nutritional risk modelling.

To systematically address these complexities, we developed the Mouth and Orofacial Health Indexing and Individualized Treatment protocol, a structured, evidence-based diagnostic and treatment framework designed for individuals with ABI, including stroke (supplementary protocol file). This protocol encompasses comprehensive assessments of oral health, orofacial function, swallowing, and nutritional status, enabling both clinical decision-making and mechanistic research. While its initial development was grounded in observational research, the present study represents an analytical advancement, applying structural equation modeling (SEM) to test the hypothesized directional pathways. While previous studies have examined the associations between stroke, oral health, oral dysfunction, dysphagia, and malnutrition in isolation [14, 15], the direct and indirect pathways linking these factors have not been evaluated. Using SEM enables simultaneous assessment of these direct and indirect relationships, thereby providing a more comprehensive understanding of the role of oral health within functional outcome models following stroke. Therefore, the primary aim of this study was to elucidate the direct and indirect pathways linking poor oral health, orofacial dysfunction, and dysphagia to malnutrition during the early stages of neurorehabilitation in stroke survivors. We hypothesized that poor oral health would exert both direct effects on malnutrition risk and indirect effects mediated through orofacial dysfunction and increased dysphagia severity. By clarifying these interrelationships, the findings may provide valuable clinical evidence to support the development of oral health-relevant, interdisciplinary interventions aimed at preventing nutritional decline in stroke survivors.

## Methods

### Participants, study design and setting

This study was embedded within the Mouth and Orofacial Health Indexing and Individualized Treatment protocol, a prospective clinical quality development project implemented at a specialized neurorehabilitation hospital i.e., Hammel Neurorehabilitation Centre and University Research Clinic (HNRC), Denmark. The current study analysis exclusively focused on stroke survivors, aligning with a theoretical framework that positions oral health as a key determinant of dysphagia and nutritional risk. All stroke survivors aged ≥ 18 years admitted to HNRC between February- September 2023 were consecutively recruited in a longitudinal observational cohort. Of the 110 screened at admission, 92 met the eligibility criteria and underwent comprehensive oral and orofacial assessments, dysphagia screening and nutritional status assessment at admission and after four weeks. Baseline assessments were performed at admission (week 1) to characterize patients’ clinical status upon entry into neurorehabilitation. The follow-up assessment at week 4 was defined to reflect the average duration of inpatient stay at our rehabilitation center, thereby representing a standardized and clinically meaningful endpoint approximating discharge for most patients. A priori sample size calculation specific was not undertaken, as the study was embedded within an ongoing clinical quality development project, as mentioned above.

**Fig. 2:**
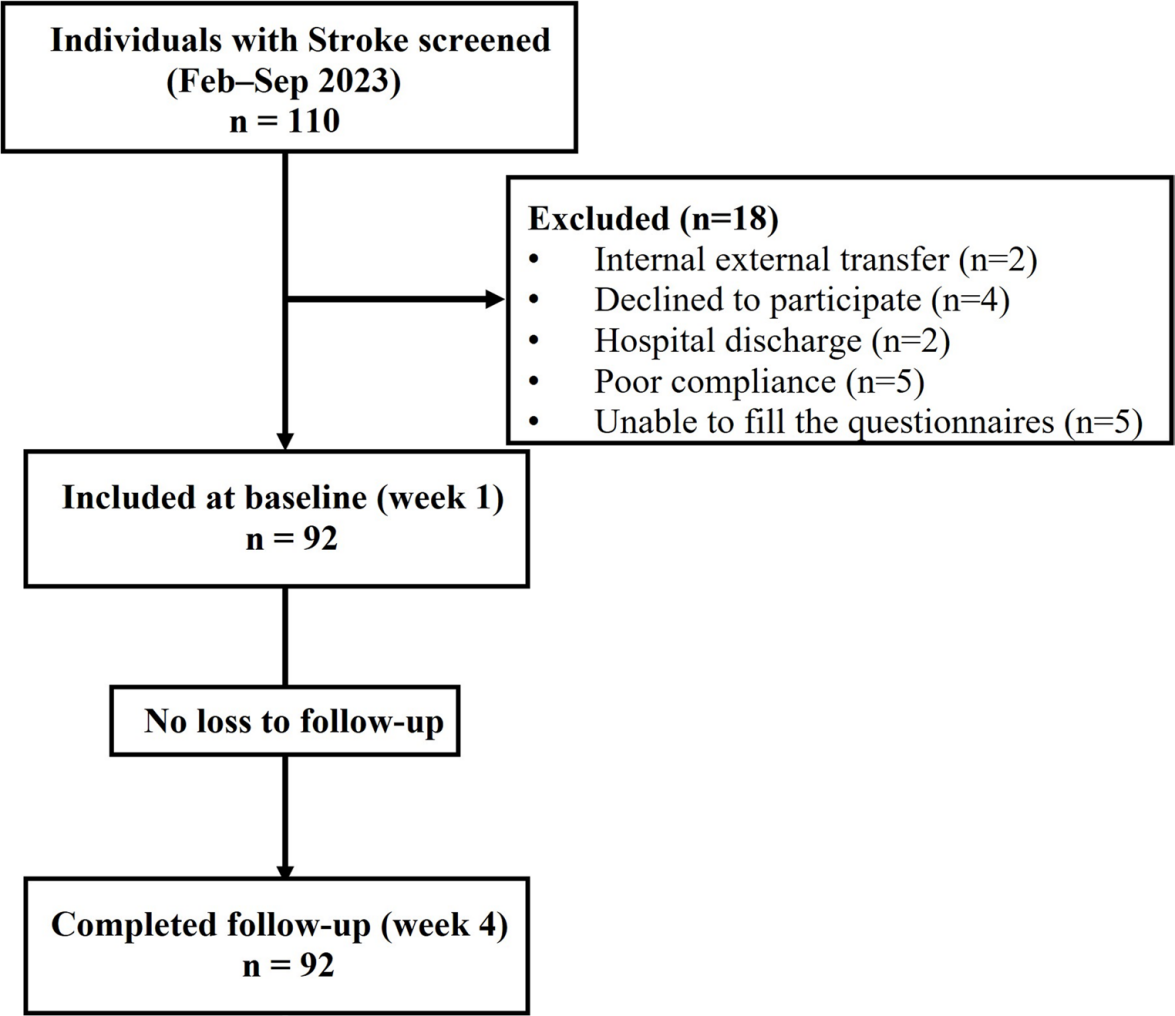
Participant flow diagram

Exclusion criteria included admission other than stroke (e.g., traumatic brain injury, neurodegenerative disorders, polyneuropathy) or pregnancy, given the potential influence of hormonal changes on oral and orofacial health [16]. Patients temporarily transferred out of HNRC for medical or logistical reasons were re-included if readmitted within five days, with the readmission date counted as day 1. Participants unable to complete assessments due to cognitive impairment, limited mouth opening, fatigue, stress, or infection were rescheduled within one week. If examination was still not possible, they were excluded. A flow diagram illustrating participant screening, eligibility, inclusion, and analysis is provided in Fig. 2.

The project was notified to the Central Denmark Region Committee on Health Research Ethics (case no. 1-10-72-124-22). In accordance with the Danish Consolidation Act on Research Ethics Review of Health Research Projects, Act no. 1338 of 1 September 2020, Sect. 14(1), the committee classified it as a “clinical quality development project,” exempting it from formal ethical approval and written informed consent. Institutional review board approval was obtained (case no. 196525). The study complied with the Helsinki Declaration II; participants received verbal and written information and were free to decline participation at any time. The study is reported in accordance with the STROBE guidelines for observational studies.

## Procedures and measures

All oral and orofacial health-related screening, clinical, and objective assessments were performed by trained dentists (SFK/MK) using standardized protocols. The same examiners conducted all assessments to ensure consistency. Prior to study initiation, both examiners were familiarized with the assessment procedures to support uniform application across participants. Demographic, socio-behavioral history, and patient information were recorded by the project nurse using standardized forms. Medical information, including primary diagnosis, comorbidities (e.g., diabetes, hypertension), brain-injury etiology, and length of stay in acute care, was extracted from electronic medical records. Complex and clinical evaluations such as dysphagia assessment, nutritional status, eating difficulties, pneumonia occurrence (defined by initiation of antibiotic therapy), body mass index and brain injury-related scores like, Early functional ability (EFA), Functional independent measures (FIM), Ranchos Los Amigos Scale (RLAS) and Functional oral intake scales (FOIS), that cover motor, cognitive, sensory, and functional domains were conducted at admission and at four weeks at HNRC by certified health professionals (nurses, physicians, occupational therapists, speech therapists, and dieticians) as part of routine rehabilitation care to monitor recovery progress [3, 10, 17]. Details are available in the supplementary protocol file.

## Dysphagia screening and clinical classification

Dysphagia was identified using the standardized multimodal swallowing assessment pathway routinely employed at HNRC. Screening was conducted within the first 24 h of admission by an occupational therapist (OT), the primary profession responsible for dysphagia management in Denmark. The screening procedure included evaluation of oral intake ability, oral secretion management, and safety of mealtime performance. Individuals who demonstrated difficulty maintaining safe, efficient, and independent oral intake were classified as having dysphagia. Comprehensive clinical swallowing examinations (F.O.T.T.–SAS) [18, 19] and instrumental assessment using Fiberoptic Endoscopic Evaluation of Swallowing (FEES) were available at the center and performed when clinically indicated. However, these assessments were not systematically applied across all participants and were therefore not required for dysphagia classification in the present study. Instead, eligibility was defined exclusively by the standardized 24-hour OT-led dysphagia screening. Accordingly, dysphagia in this study reflects clinically assessed swallowing impairment based on standardized routine screening rather than instrumentally confirmed diagnosis, which aligns with established multidisciplinary assessment pathways [18, 19].

## Nutritional outcome and oral frailty

Nutritional status was assessed using the Mini Nutritional Assessment (MNA), a validated ‘screening’ and ‘assessment’ tool combining anthropometric data, dietary intake, and functional indicators to classify individuals as well-nourished, at risk of malnutrition, or malnourished [20]. Oral frailty was evaluated using the Oral Frailty Index-8 (OFI-8), an eight-item questionnaire assessing oral health-related behaviors and functions, with total scores ranging from 0 to 11 and higher scores indicating greater frailty [21]. Details are available in the supplementary protocol file.

## Comprehensive oral health assessment

The modified Bedside Oral Examination (mBOE) was used as the primary screening tool, comprising both oral health and orofacial health components [22]. Following screening, a standardized full-mouth examination recorded DMF-T index, plaque, bleeding on probing (BoP), tongue coating index (TCI) [23], halitosis [24] and unstimulated salivary flow rate [25] (graded as normal < 33%, moderate 34–66%, or severe > 66%) according to standardized and previously validated clinical protocols. Details are available in the supplementary protocol file.

### Comprehensive orofacial function assessment

Orofacial function was assessed using a combination of motor, sensory, and strength-based measures representing established domains of oral functional performance. Oral motor function was assessed using the PaTaKaRa™ app to measure fine motor speed and coordination of lips, tongue, and cheeks [26]. Bite force, representing masticatory strength, was measured with a handheld strain gauge-based force meter [27], and oral sensory function via oral stereognosis [28]. Additional measures included tongue/lip pressure (Iowa Oral Performance Instrument) [29], peak expiratory flow rate [30] and masticatory performance with color-changeable gum [31], all of which are widely applied in oral function research and established methods for clinical assessment [32]. Details are available in the supplementary protocol file.

### Statistical analysis

Descriptive analyses were performed to compare subgroups stratified by dysphagia at admission. Categorical variables were analyzed using Fisher’s exact test, and continuous variables using the Wilcoxon test.

**Fig. 3:**
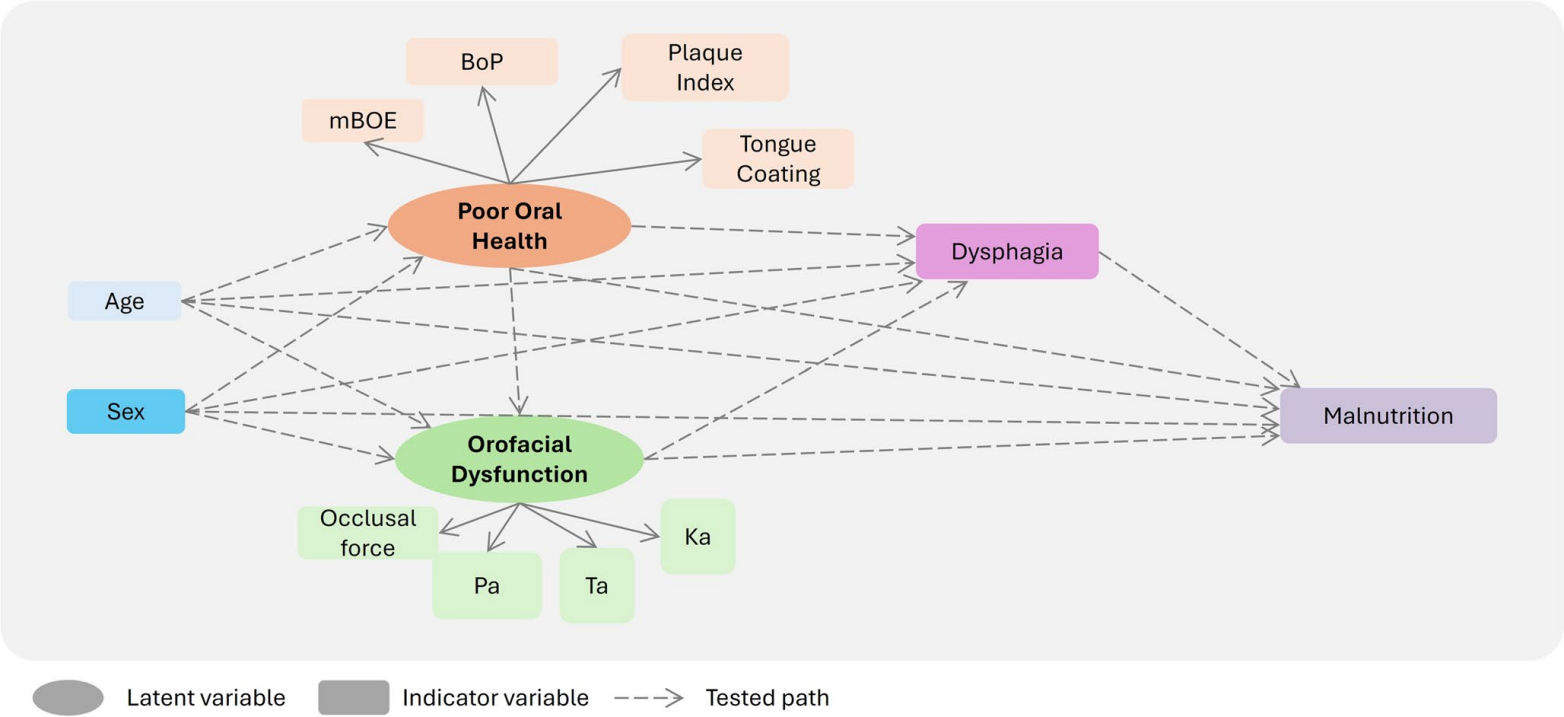
Theoretical model of hypothesized relationships among study variables mBOE: modified bedside oral examination

To empirically evaluate the hypothesized directional pathways linking poor oral health, orofacial dysfunction, dysphagia, and malnutrition risk, structural equation modeling (SEM) was conducted. The conceptual framework underpinning the structural equation models (Fig. 3) was based on prior observational findings and theoretical models positioning [33, 34] on oral health as a determinant of swallowing and nutritional function. Poor oral health and orofacial dysfunction were modeled as latent variables, while dysphagia and malnutrition risk (measured by MNA at week 4) were specified as observed outcomes. Age and sex were included as covariates to adjust for potential confounding. Although additional clinical variables (for example, FIM, FOIS, and RLAS) were available, only age and sex were included in the SEM to preserve model stability given the sample size.

The latent construct of poor oral health was defined by the shared variance among mBOE, BoP, plaque index, and TCI, whereas orofacial dysfunction was derived from bite force and ODK measures for Pa, Ta, and Ka syllables in the model (SEM). The specification of latent constructs was guided by a conceptual framework grounded in prior literature, in which oral health and orofacial function are considered multidimensional determinants of swallowing and nutritional outcomes. Indicators were initially selected based on their theoretical relevance and clinical interpretability. Exploratory factor analysis was subsequently used as a complementary step to assess the empirical coherence of these constructs and to identify indicators that contributed meaningfully to the shared variance of each latent variable. Indicators with low factor loadings (< 0.40) and limited conceptual alignment were excluded to improve construct validity and model parsimony, rather than to optimize statistical fit. This iterative process aimed to balance theoretical plausibility and empirical adequacy, consistent with recommended practices in latent variable modeling [33, 34]. While alternative model specifications were explored during model development, the final model was selected based on consistency with the underlying theoretical framework, construct interpretability, and overall model stability, rather than solely on statistical fit indices.

Model estimation was then performed using the weighted least squares mean and variance adjusted (WLSMV) method to account for ordinal and non-normally distributed data. This estimator provided robust control of residual variances and enabled full information estimation to address missing data. Missing data were imputed using maximum likelihood estimation to retain all available information. Model fit was evaluated using standard indices, including the root mean square error of approximation (RMSEA), comparative fit index (CFI), Tucker–Lewis index (TLI), and standardized root mean square residual (SRMR). Thresholds of RMSEA < 0.08, CFI ≥ 0.95, and TLI ≥ 0.90, and SRMR < 0.08 were considered indicative of good model fit. A non-significant chi-square test was interpreted as indicating no substantial discrepancy between the model and the observed data. Direct and indirect path coefficients were reported as standardized estimates (β), along with standard errors (SE) and p-values. Indirect effects were estimated using the product of coefficients method, with significance tested by the delta method. Indirect effects with p-values ≥ 0.05 were interpreted as non-significant and considered exploratory. A two-tailed p-value < 0.05 was considered statistically significant. Model estimation was performed using Mplus 7.0 software.

## Results

### Descriptive and subgroup comparisons

The study population comprised of 92 individuals with a mean age of 59.7 years, predominantly men (70.7%). The mean BMI was 26.2 kg/m². On average, the onset of brain injury occurred 34.1 days before admission to the rehabilitation center, following a mean stay of 16.3 days in the acute care setting. Dysphagia was identified in 46.7% of participants, and 21.8% reported eating difficulties. Hypertension was highly prevalent (66.3%), while diabetes was present in 19.6% of the sample. Regarding smoking status, half of the participants were former smokers (50.0%), 35.4% had never smoked, and 14.6% were current smokers (Supplementary Table 1). Comprehensive baseline demographic and clinical characteristics, including measures of stroke severity, functional status, and other relevant variables, are presented in Supplementary Table 1.

Table 1 presents the baseline characteristics of the sample, stratified by dysphagia status. These subgroup comparisons are descriptive and provide clinical context, whereas dysphagia is modeled as a mediator within the structural equation framework. Patients with dysphagia had markedly poorer nutritional, oral, and orofacial health profiles compared to those without dysphagia. Malnutrition was also more prevalent among dysphagic individuals (72.2% vs. 37.7%, *p* = 0.011). According to the OFI-8, a high risk of oral frailty was substantially more frequent in the dysphagic group (60.0% vs. 13.2%, *p* = 0.001). The feeding route distribution also differed significantly (*p* < 0.001), with nearly all non-dysphagic individuals were on an oral diet (97.9%).

**Table 1:** Baseline parameters in stroke survivors and subgroups stratified by dysphagia status

	Total N (%)	Non-dysphagic N (%)	Dysphagic N (%)	P-value
Malnutrition Parameters				
Mini Nutritional Screening				0.011
Normal	1 (1.8)	1 (2.5)	-	
At risk	29 (50.9)	25 (62.5)	4 (23.5)	
Malnourished	27 (47.4)	14 (35.0)	13 (76.5)	
Malnutrition Indicator Score				0.351
Normal	12 (21.4)	10 (25.6)	2 (11.8)	
At risk	38 (67.9)	26 (66.7)	12 (70.6)	
Malnourished	6 (10.7)	3 (7.7)	3 (17.6)	
Oral Frailty Index-8				0.001
Low risk	26 (44.1)	22 (55.0)	4 (21.1)	
Moderate risk	15 (25.4)	12 (30.0)	3 (15.8)	
High risk	18 (30.5)	6 (15.0)	12 (63.2)	
Feeding status				<0.001
Oral	67 (72.8)	47 (97.9)	18 (42.9)	
Nasal	15 (16.3)	1 (2.1)	14 (33.3)	
PEG	10 (10.9)	-	10 (23.8)	
	Mean (SD)	Mean (SD)	Mean (SD)	
Oral Health Parameters				
mBOE score	11.1 (3.1)	10.1 (2.4)	12.4 (3.7)	0.006
Bleeding on probing	67.8 (22.2)	59.0 (19.8)	73.9 (22.6)	0.023
Plaque index	70.7 (18.6)	66.1 (17.1)	74.4 (19.6)	0.082
Tongue coating score	16.2 (5.2)	15.2 (4.6)	17.5 (6.1)	0.184
Halitosis score				0.015
Grade 1	21 (22.8)	14 (29.2)	6 (14.3)	
Grade 2	46 (50.0)	27 (56.2)	19 (45.2)	
Grade 3	25 (27.2)	7 (14.6)	17 (40.5)	
Orofacial Health Parameters				
Posterior occlusal contacts	6.2 (2.4)	6.7 (2)	5.5 (2.8)	0.020
Bite force	0.3 (0.2)	0.4 (0.2)	0.3 (0.2)	0.005
Oral diadochokinesis				
Pa	4.7 (1.8)	5.4 (1.3)	3.6 (2.0)	<0.001
Ta	4.1 (1.8)	4.7 (1.6)	3.1 (1.7)	<0.001
Ka	3.7 (1.9)	4.4 (1.5)	2.6 (1.9)	<0.001
Oral stereognosis	6.6 (4.3)	8.5 (3.7)	3.8 (3.6)	<0.001
Tongue pressure	32.1(14.7)	38.2 (12.4)	24.0 (13.8)	<0.001
Lip pressure	12.5 (8.5)	13.8 (10.1)	10.5 (5.2)	0.423
Peak Exploratory Flow	243.1 (151.7)	314.6 (141.9)	145.6 (99.9)	<0.001
Forced Expiratory Volume	2.0 (0.9)	2.4 (0.7)	1.4 (0.8)	<0.001
Masticatory performance	3.0 (1.2)	3.4 (1.1)	2.4 (1.2)	<0.001

*PEG* Percutaneous Endoscopic Gastronomy, *mBOE* modified bedside oral examination

Most individuals receiving nutrition via nasogastric tube (33.3%) or PEG (23.8%) were dysphagic. Regarding oral health, dysphagic participants presented higher mBOE scores (12.4 vs. 10.1, *p* = 0.006) and higher gingival bleeding on probing (73.9% vs. 59.0%, *p* = 0.023). Halitosis scores indicated a greater proportion of dysphagic patients in the worst category (score 3: 40.9% vs. 14.3%, *p* = 0.015). Orofacial function measures were consistently lower in the dysphagic group, including bite force (0.3 vs. 0.4, *p* = 0.005), oral diadochokinesis for Pa (3.6 vs. 5.4, *p* < 0.001), Ta (3.1 vs. 4.7, *p* < 0.001), and Ka (2.6 vs. 4.4, *p* < 0.001), oral stereognosis (3.8 vs. 8.5, *p* < 0.001), tongue pressure (24.0 vs. 38.2, *p* < 0.001), peak exploratory flow (145.6 vs. 314.6, *p* < 0.001), forced expiratory volume (1.4 vs. 2.4, *p* < 0.001), and masticatory performance (2.4 vs. 3.4, *p* < 0.001). In addition, the number of posterior occlusal contacts was significantly lower among dysphagic individuals compared to non-dysphagic participants (5.5 ± 2.8 vs. 6.7 ± 2.0, *p* = 0.020).

### Structure equation modeling (SEM)

In the SEM framework, dysphagia was modeled as an intermediate (mediating) variable linking orofacial function and nutritional outcomes. The measurement model showed an acceptable fit to the data, with χ² = 70.385 (df = 54, *p* = 0.066), RMSEA = 0.062 (90% CI: 0.000–0.100; *p* = 0.294 for RMSEA ≤ 0.05), CFI = 0.957, TLI = 0.933, and SRMR = 0.075. These indices collectively indicate good model fit. All latent variables formed satisfactory constructs with convergent factorial loadings. All indicators of poor oral health showed factor loadings equal to or higher than 0.5 and p-values < 0.001, while all indicators of orofacial dysfunction had factor loadings above 0.4 and p-values < 0.001, supporting the internal consistency of both constructs (Table 2).

**Table 2:** Standardized factor loadings, standard errors, and p-values for the latent variables poor oral health and orofacial dysfunction (*n* = 92)

Latent variable	Indicator	Factor loading	Standard error	*p*-value
Poor Oral Health	Bleeding on Probing	0.573	0.082	< 0.001
	Plaque Index	0.523	0.086	< 0.001
	mBOE Score	0.961	0.068	< 0.001
	Tongue Coating Score	0.614	0.083	< 0.001
Orofacial Dysfunction	Bite Force	0.447	0.097	< 0.001
	Pa	0.742	0.057	< 0.001
	Ta	0.903	0.030	< 0.001
	Ka	0.938	0.026	< 0.001

*mBOE* modified bedside oral examination

**Fig. 4:**
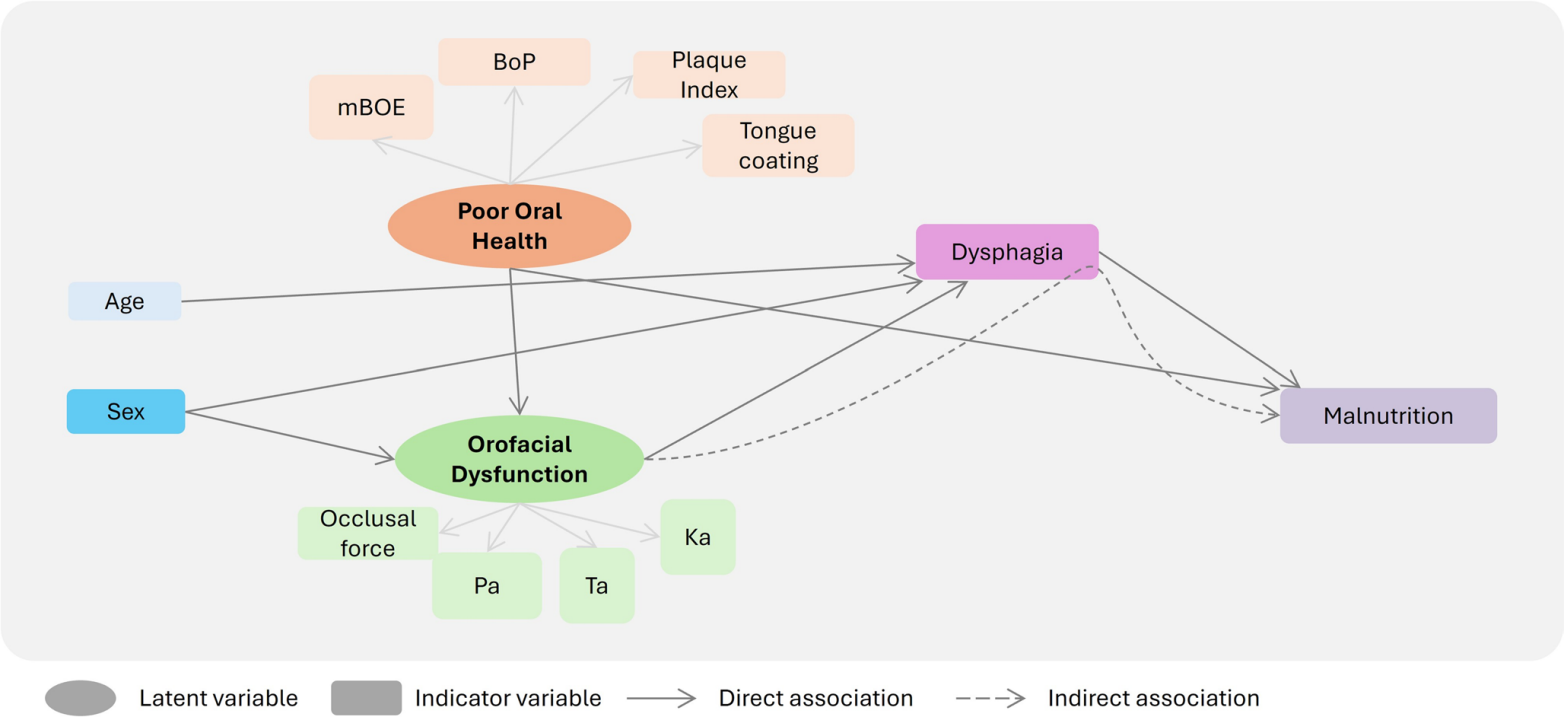
Significant pathways identified in the Structural Equation Model mBOE: modified bedside oral examination

Table 3 shows the results of the direct pathways evaluated by SEM. Age and sex were retained as covariates in the SEM model, and their path estimates are reported in Table 3 but are not interpreted as primary findings. Poor oral health was negatively associated with orofacial dysfunction (β = -0.407, SE = 0.111, *p* < 0.001) and positively associated with malnutrition risk (β = 0.333, SE = 0.146, *p* = 0.023). Orofacial dysfunction was negatively associated with dysphagia severity (β = -0.505, SE = 0.096, *p* < 0.001), and dysphagia was positively associated with malnutrition risk (β = 0.307, SE = 0.143, *p* = 0.031). The significant pathways are shown in Fig. 4.

**Table 3:** Structural regression coefficients for direct pathways between exposure and outcome (*n* = 92)

Exposure variable	Outcome variable	β	SE	*p*-value
Sex	Poor Oral Health	-0.179	0.105	0.090
	Orofacial Dysfunction	-0.250	0.103	0.015
	Dysphagia Severity	-0.205	0.089	0.021
	Risk of malnutrition	-0.064	0.131	0.628
Age	Poor Oral Health	0.174	0.104	0.094
	Orofacial Dysfunction	-0.087	0.106	0.408
	Dysphagia Severity	0.233	0.086	0.006
	Risk of malnutrition	-0.213	0.119	0.073
Poor Oral Health	Orofacial Dysfunction	-0.407	0.111	< 0.001
	Dysphagia Severity	0.077	0.103	0.454
	Risk of malnutrition	0.333	0.146	0.023
Orofacial Dysfunction	Dysphagia Severity	-0.505	0.096	< 0.001
	Risk of malnutrition	0.033	0.176	0.850
Dysphagia Severity	Risk of malnutrition	0.307	0.143	0.031

*β* standardized coefficient, *SE* standardized error

Table 4 presents the specific indirect pathways. The indirect pathway from orofacial dysfunction to malnutrition through dysphagia was at the threshold of statistical significance (β = −0.155, SE = 0.079, *p* = 0.050). No statistically significant indirect pathway was observed from poor oral health to malnutrition through orofacial dysfunction and dysphagia (β = 0.063, SE = 0.037, *p* = 0.091).

**Table 4:** Specific indirect pathways on risk of malnutrition

Indirect pathway	β	SE	*p*-value
Orofacial Dysfunction → Dysphagia → Malnutrition	-0.155	0.079	0.050
Poor Oral Health → Dysphagia → Malnutrition	0.024	0.033	0.473
Poor Oral Health → Orofacial Dysfunction → Malnutrition	-0.013	0.072	0.851
Poor Oral Health → Orofacial Dysfunction → Dysphagia → Malnutrition	0.063	0.037	0.091

*β* standardized coefficient, *SE* standardized error

## Discussion

This study used descriptive, longitudinal, and SEM analyses to explore the interrelationships between poor oral health, orofacial dysfunction, dysphagia, and malnutrition in stroke survivors. Rather than examining these factors in isolation, the present work clarifies how they interact within a coherent pathway contributing to nutritional vulnerability during rehabilitation. Findings suggest that poor oral health is associated with malnutrition risk, with evidence supporting a direct association and pathways involving orofacial dysfunction and dysphagia, emphasizing its role in oral-systemic functional decline during post-stroke recovery. From an oral-systemic perspective, these results provide conceptual support for considering oral health and orofacial function as integral components of malnutrition risk assessment in stroke rehabilitation.

### Oral health as a foundational determinant

Our model revealed that poor oral health was significantly associated with orofacial dysfunction, which in turn was associated with dysphagia severity. This supports a conceptual framework in which the functional and structural consequences of suboptimal oral health, such as gingival inflammation, discomfort, microbial load, and mucosal damage, may contribute to oral muscular coordination and sensory feedback [35]. These findings resonate with prior studies linking oral health status to sarcopenia of the orofacial muscles and with emerging work showing that edentulism, dry mouth, and biofilm burden are associated with swallowing difficulties in older adults [36, 37]. Clinically, these findings imply that improving oral health may potentially reduce local symptoms perhaps preserve orofacial motor capacity, thereby supporting safer and more efficient eating and swallowing. Such mechanisms are directly relevant to clinical dentistry and oral rehabilitation, with potential implications for maintaining oral intake, improving dietary adequacy, and ultimately preventing or slowing nutritional decline [38, 39].

The latent variable representing oral health was constructed from clinically relevant and widely validated indicators, including mBOE score, gingival bleeding on probing, plaque index, and tongue coating. Notably, the strong internal consistency of this construct (all factor loadings > 0.5, *p* < 0.001) accentuates the suitability of these indicators for inclusion in interdisciplinary screening protocols, such as the model used here.

### Orofacial dysfunction as a key mediator

Orofacial function, composed of bite force and diadochokinetic syllable repetition rates (/pa/, /ta/, /ka/), emerged as a statistically and theoretically robust mediator between orofacial health and dysphagia. Bite force represents masticatory strength, while diadochokinesis reflects neuromuscular coordination of the lips and tongue, both critical for bolus preparation and safe swallowing initiation. The observed association between poor oral health and orofacial dysfunction may reflect both inflammatory pathways and disuse-related deconditioning [40, 41].

The relationship between orofacial dysfunction and dysphagia severity supports their integration into interdisciplinary assessments for post-stroke dysphagia. Our results further showed that orofacial dysfunction did not show a statistically significant indirect effect, although the estimates were consistent with a potential mediating role of dysphagia, suggesting that orofacial dysfunction may contribute to malnutrition primarily through its impact on swallowing.

### Dysphagia and nutritional risk: confirming the cascade

Consistent with the previous literature, dysphagia was directly associated with malnutrition [42, 43], reinforcing the clinical necessity of early dysphagia screening and intervention. Nutritional status, however, is a multifactorial construct shaped by medical, functional, and psychosocial determinants. Further, difficulties in eating, chewing, and swallowing constitute only one component of the overall risk profile [39, 44, 45] and may be important “contributors” to nutritional risk [46, 47]. However, the absence of a direct path between orofacial dysfunction and malnutrition combined with an indirect path via dysphagia did not reach statistical significance which emphasizes the importance of evaluating swallowing as the key mediator linking orofacial dysfunction to nutritional outcomes [36, 44, 48]. For nutrition practitioners, incorporating oral health and orofacial assessments into dysphagia screening could help identify patients at nutritional risk earlier, enabling timely interventions such as texture modification, supplementation, and targeted oral rehabilitation [49].

Interestingly, we also identified a significant direct effect of poor oral health on malnutrition, independent of dysphagia. This could reflect additional pathways not directly modeled in our study, including oral pain, xerostomia, taste alterations, reduced appetite, and microbial dysbiosis [50, 51]. These oral and orofacial signs and symptoms may limit food intake even in the absence of clinically overt dysphagia and should be explored in future research using patient-reported outcome measures.

### Clinical and translational implications

The multifactorial nature of stroke rehabilitation demands a thorough and interdisciplinary approach, and the results of this study provide empirical validation for the structure and logic of the protocol, which emphasizes early identification of oral health deterioration and orofacial dysfunction in post-stroke individuals. In practice, our findings could inform the development of enhanced nutrition screening protocols that include oral health parameters, aligning with international guidelines (ESPEN, ASPEN) that call for holistic malnutrition risk assessment. Such integration could improve prediction, prioritization, and personalization of nutritional interventions [52].

The clinical utility of the model is high. For instance, the presence of a high plaque index and poor tongue mobility could flag a patient as being at risk for later malnutrition. For instance, hospitalized patients with elevated plaque scores were significantly more likely to exhibit moderate to severe malnutrition, while community-dwelling older individuals with suboptimal tongue pressure demonstrated markedly increased odds of malnutrition [53], prompting proactive dietary counselling, oral rehabilitation, or nutritional supplementation. Moreover, our findings suggest that interventions targeting oral health may be associated with downstream improvements in swallowing and nutritional status, although this requires confirmation in interventional studies. Although pneumonia was not included in the final SEM due to limited cases and poor model fit, it remains a clinically important outcome potentially influenced by the oral–swallow–nutrition axis. Future studies with larger samples may incorporate pneumonia into their analytical model. A conceptual illustration or brief descriptive exploration could be useful to depict how the current findings may relate to infection risk and respiratory complications.

### Limitations and future directions

The sample size (*N* = 92) is relatively modest for SEM involving multiple latent constructs and may affect model stability, precision, and the ability to detect smaller effects. Therefore, the findings should be interpreted as exploratory, as the absence of positive associations might have resulted from an underpowered sample. However, it is unlikely that positive associations would change, as an increased sample size should narrow down their respective confidence intervals. The SEM was adjusted for age and sex; however, additional clinical variables, including stroke severity and functional status measures, were not incorporated because of sample-size and model-stability constraints. Therefore, residual confounding cannot be excluded, and age- and sex-related path estimates should not be interpreted as primary findings. Although the model fit indices (RMSEA = 0.062, CFI = 0.957, TLI = 0.933) indicate an excellent fit, the relatively small sample size may limit the power to detect weaker indirect effects and interaction terms. A larger sample would allow for multi-group comparisons (for example, by sex, age, or oral status) and permit inclusion of more granular outcome measures. Another limitation is the cross-sectional nature of oral health and orofacial assessments, whereas nutritional risk was evaluated longitudinally. The selection of week 4 as the follow-up time point reflects the typical duration of inpatient rehabilitation in our setting, allowing standardized assessment across participants; however, variation in individual length of stay may influence outcome trajectories. Although this design supports temporal ordering, causal inferences remain limited. Future studies should incorporate repeated measures over time to capture dynamic trajectories. Additionally, patient-reported outcomes, such as pain during chewing, xerostomia, and self-rated appetite, were not included in the present model but may explain the direct effect of oral health on nutrition. In the current study, dysphagia classification was based on standardized clinical screening rather than systematic instrumental assessment, which may introduce misclassification bias. Future studies incorporating systematic instrumental assessment would enhance diagnostic precision and strengthen model validity. Finally, this study was conducted in a specialized rehabilitation setting, and the generalizability of findings to acute stroke units, community-based settings, or other neurological populations should be tested in future validations of the protocol.

## Conclusions

This study provides novel evidence of associations between poor oral health and malnutrition risk in stroke survivors via reduced oral motor function and dysphagia. These findings support the multidimensional assessment approach embedded in the protocol and emphasize the importance of interdisciplinary oral care as an integral component of stroke rehabilitation. Structural equation modeling proved an effective method to explore these complex interactions, providing a framework for future hypothesis-driven and interventional research. Future research should extend and validate this model across diverse clinical populations and settings.

## Supplementary Information

Below is the link to the electronic supplementary material.

Supplementary Material 1

## Data Availability

The data in the current project are defined as sensitive personal data. The data cannot be shared publicly due to existing data protection laws in Denmark imposed by the Danish Data Protection Agency. The access may be granted on anonymized data and on a case-by-case basis after approval from the PI and the research group.
