# Supplementary material for: Oral health and its association with dysphagia severity and nutritional vulnerability after stroke: a structural equation modeling study
Source: Clin Oral Investig. 2026 May 28;30(6):252. doi: 10.1007/s00784-026-06951-3 (PMC13219075; doi:10.1007/s00784-026-06951-3)
Supplement: Supplementary file 1 — Supplementary Material 1 [file 784_2026_6951_MOESM1_ESM.doc]

**Data collection sheet**

**Mouth and Orofacial Health Indexing & individualized Treatment plan**

**WEEK: 1&4**

***Initials of Patient Name****:*

***Date of examination****:*

***Unique ID* (patient initials + ward number + date of admission at HNRC)*:***

______________________________________

**Write reasons of missing data in below instrument, wherever required. For example:**

**1) Patient irresponsive**

**2) Data absent in patient journal**

**3) Mention, any other reason, if any**

**Mouth and Orofacial Health Indexing & individualized Treatment plan**

- ***Demographics:***

***Name/ ID****: ______________________________* ***Assessment week****: week 1/ week 4*

***Age****:__ yrs.* ***Gender****:* *Male /Female* ***Type & name of ward:*** *____HS / ___Region ward*

***Weight:****_______* ***Height****:_____________* ***BMI:****______________* ***Date of BMI:_________***

- ***Relevant medical information:***

***Main diagnosis and code:****________________* ***Length of stay in acute care:****___________ days*

***Date of admission at Hammel:****________________****Date of brain injury:****_______________* ***Feeding status:*** *Oral/ Tube (PEG/IV/ Nasal)/ Oral & tube*

***Dysphagia (DR139):*** *Yes/ No* ***Tracheostomy tube:*** *Yes/No*

***Pneumonia (In last 12 months): Yes/ No, If yes, Date:_________________________________***

***Hypertension:*** *Yes/ No*  ***Diabetes:*** *Yes/ No*

- ***Social and behavioral history:***

1. ***Level of Education****:*

Basic (1-9 years)***/*** High school (9-12 years)***/*** Vocational***/*** Technical degree***/*** Higher Education***/*** University degree/ …………………………………….. (other)

1. ***Smoker:*** *Never/ Former/ Current*
2. ***Dental behavioral History (Before hospitalization)***

***Tooth brushing frequency****: Once/ twice/ thrice/ otherwise stated*

***Type of toothbrush:*** *Manual* ***(small head or normal)****/ Electric/ Both*

***Regular Dental Visit* (*In the last 12 months):*** *No/ once/ twice****/ otherwise mentioned***

**Step 1: Screening**

**modified Bedside Oral Examination (mBOE) screening tool**

**using tongue depressor & torch**

1. **Oral health**


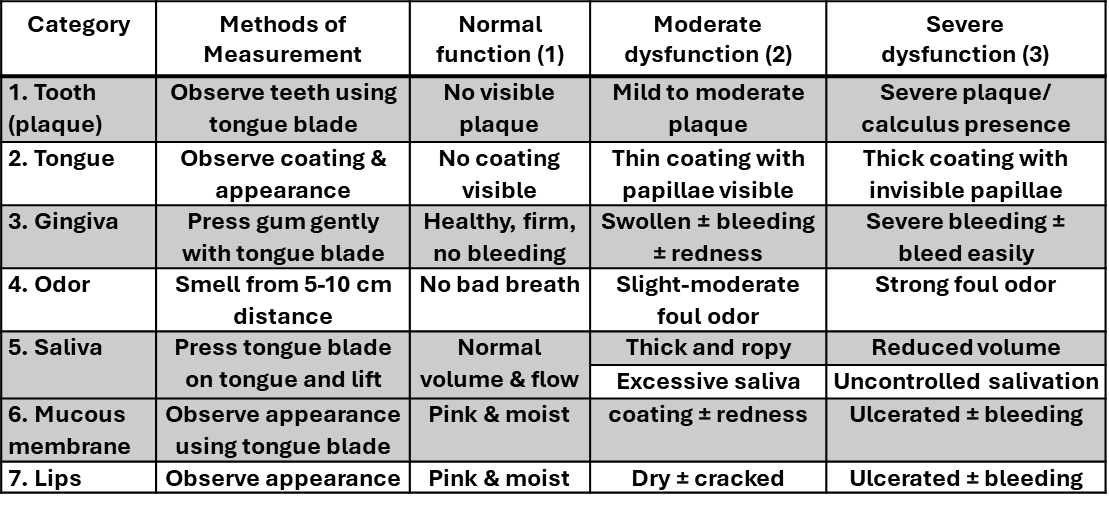


1. **Orofacial Health (Associated risk factors to oral care)**

**
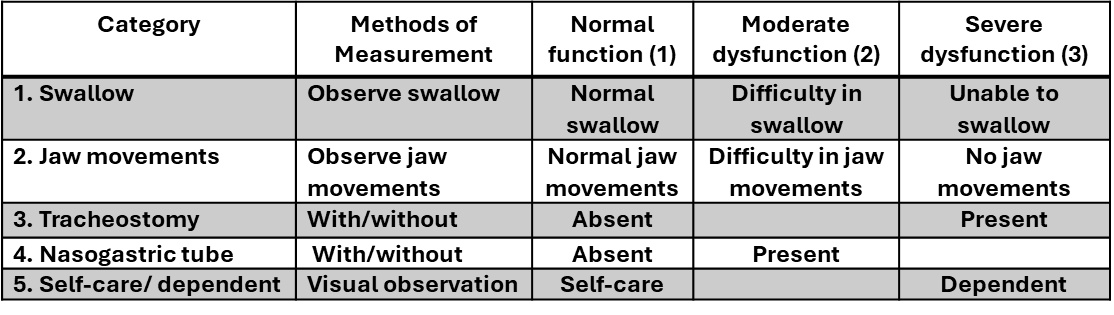
**

**Step 2: Clinical assessment using UNC-15 probe, mouth mirror and arch explorer**

1. **Oral Health**
2. ***Decayed, missing and filled tooth examination (DMFT examination)***

**Write, D= Decay; M=Missing; I= IMPLANT; F=Fixed prosthesis; R=Restoration; T= Temporory/ removable prosthesis) in the affected tooth column**

**
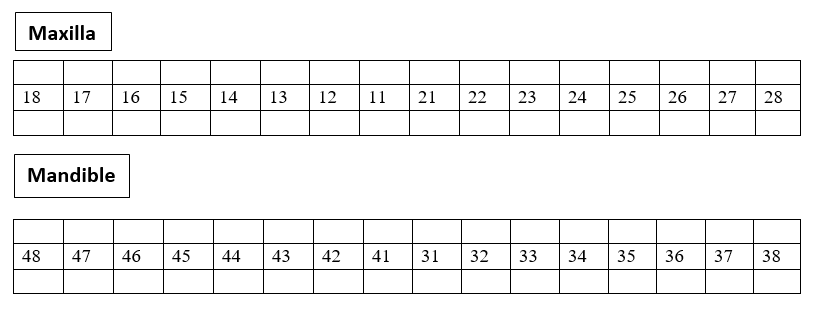
**

**Number of posterior contacts (all tooth including natural, pontics and implants but excluding removable prosthesis after canine. In ideal situation it will be 8 contacts in entire oral cavity, which is two premolars and two molars contacts on each side):**

**Duration of prosthesis:_____________ in months.**

1. ***Supra-gingival plaque and calculus examination (UNC-15 probe)***


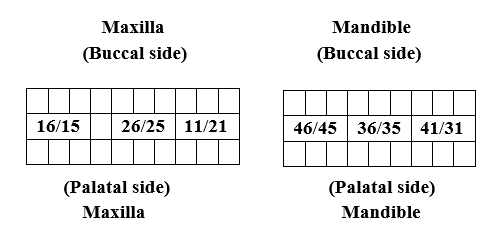
**Scoring system:** At and 1 mm below free gingival margin (FGM) and attached tooth surface.

No visible plaque: 1

Presence of plaque found by instrumental evaluation: 2

Presence of abundant plaque/ trace of calculus which is visible by naked eye: 3

Plaque/ calculus indexing: 6 sites each tooth x 6 tooth x 1-3 (min.-max. score): 36-108

Total indexing: Total score (36-108)/ 108 x 100 = ________ %

Grading: 0-33% normal; 34-66: moderate; >66: severe

1. ***Periodontal pocket depth (PD) measurement (mm)***


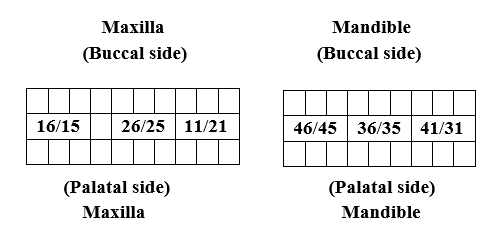
The distance from the FGM to the bottom of the sulcus/ pocket. **Write PD in mm.**

Note: If the first molar is missing in any quadrant, then **second premolar** will be examined. In case, right/ left central incisor is missing then neighboring central incisor will be examined.

1.
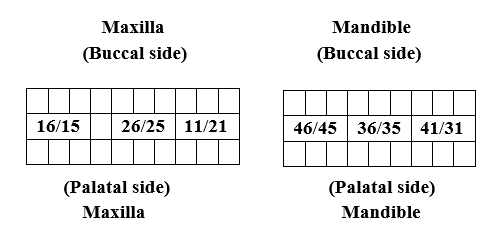
***Bleeding on probing examination***

Scoring system: UNC-15 probing in periodontal pocket and wait till 10 sec.

No bleeding: 1

Bleeding: 2

Severe bleeding: 3

Calculation: 6 sites each tooth x 6 tooth x 1-3 (min.-max. score): 36-108

Total indexing: Total score (36-108)/ 108 x 100 = ________ %

0-33% normal; 34-66: moderate; >66: severe

1. ***Tongue coating index***

**No coating: 1 (Tongue coating not visible).**

**Coating: 2 (Thin coating, papillae of tongue visible).**

**Severe coating: 3 (Tongue coating very thick, papillae of tongue not visible).**

**Tongue coating index (TCI):**

**Total score (9-27)/ 27 x 100 = ________%.**

0-33% normal; 34-66: moderate; >66: severe

1.
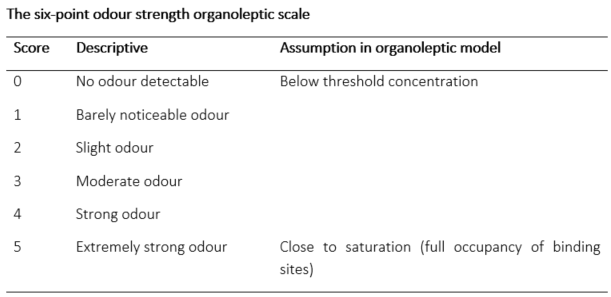
***Organoleptic Assessment (OLS)***

Participants are upright or reclined if in bed and instructed to relax with their head slightly backwards. They are instructed to close their mouth and breathe through their nose and hold the breath in their mouth for up to **1 min** whilst breathing through their nose. The participants are then be asked to open their mouth whilst the organoleptic judge approaches with his/her nose approximately 5–10 cm away from the participant’s mouth. This sample are rated according to the six-point scale.

1. ***Saliva examination (unstimulated): Cotton roll method***

**Time required 2 min for saliva collection.**

**Material required: Cotton rolls tied with floss, disposable cups, weighing scale, timer.**

1. Weigh the disposable cup with three sterile cotton rolls and note the weight X.

2. The timing starts, after the examiner place three sterile cotton rolls into patients mouth between the cheek and the upper molars (both sides) as well as under the tongue.

3. After 2 minutes, all 3 cotton rolls dipped in saliva are taken out and is placed in a disposable cup.

4. **Volume of saliva** collected is determined by calculation of the difference between the weight (± 1 mg) of the **disposable container** before and after sampling, where 1 mL is equivalent to 1g.

5. **Flow rate**: divide the volume of saliva collected by the time taken for collection.

|  | Item | Weight before saliva collection | Weight after saliva collection | Actual volume  (1 ml = 1 gm) |
| --- | --- | --- | --- | --- |
| 1 | Volume of saliva |  |  |  |
| 2 | **Salivary flow rate** | Actual volume/ actual time taken for saliva collection (2 min) | |  |

**FOR HYPOSALIVATION**

**Normal salivary flow rate: 0.3 - 0.5 ml/ min**

**Moderate dysfunctional salivary flow rate: 0.1 - 0.299 ml/ min**

**Severe dysfunctional salivary flow rate: <0.1 ml/ min**

**FOR HYPERSALIVATION**

**Normal salivary flow rate: 0.3 - 0.5 ml/ min**

**Moderate dysfunctional salivary flow rate: 0.5 - 1.0 ml/ min**

**Severe dysfunctional salivary flow rate: >1.0 ml/ min**

1. **Orofacial Health (Associated risk factors to oral care)**
2. ***Dysphagia screening: YES/ NO***

| **Trials** | **Pa** | **Ta** | **Ka** |
| --- | --- | --- | --- |
| 1. |  |  |  |
| 2. |  |  |  |
| Maximum output |  |  |  |

1. ***Eating difficulties screening: YES/ NO***

| **Shape** | **Scores (0/1/2)** | |
| --- | --- | --- |
| Circle and ellipse |  |  |
| Square and rectangle |  |  |
| Triangle and semicircle |  |  |

1. ***Oral (Pa-Ta-Ka) motor function:***
2. ***Oral sensory function (stereognosis):***

0: For not identifying the test sample.

1: For incorrect identification within the same group of forms.


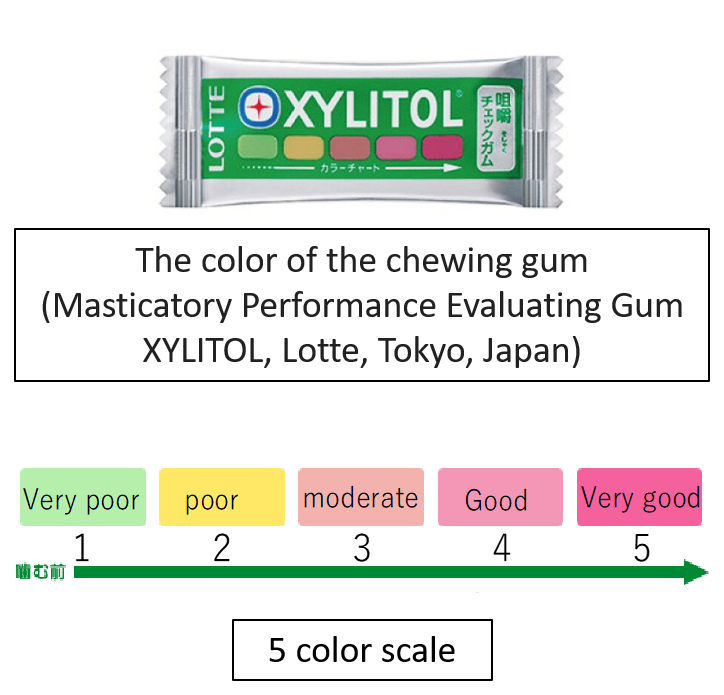
2: For correct identification of the test sample.

1. ***Masticatory performance & EMG record:***

The participants is instructed to chew the chewing gum for 60 seconds. The masticatory performance is evaluated by measuring changes in the color of the gum using a color scale.

| Trials | Tongue pressure | Lip pressure |
| --- | --- | --- |
| 1 |  |  |
| 2 |  |  |
| Maximum output |  |  |

1. ***Tongue-lip pressure***

| Trials | PEF | FEV |
| --- | --- | --- |
| 1. |  |  |
| 2. |  |  |
| Maximum output |  |  |

1. ***Peak expiratory flow (PEF)***
2. ***Occlusal force***

| Trials | Bite force recording |
| --- | --- |
| 1. |  |
| 2. |  |
| Maximum output |  |

1. ***Brain injury related measurement scores and sub scores (Medical Journal):***

|  | **Total**  **Score** | **Oral and Dysphagia related sub scores**  **Date of score** | | | | |
| --- | --- | --- | --- | --- | --- | --- |
| **FIM** |  | Eating | Motor and Cognitive | Understanding | Expression | Memory |
|  |  |  |  |  |
| **RLAS** |  |  | | | | |
| **FOIS** |  |  | | | | |
| **EFA** |  | Orofacial-stimulation | | Swallow | Tongue | Mimic |
|  | |  |  |  |

1. Oral fraility index (OFI-8)

|  | Items of Oral Frailty | YES | NO |
| --- | --- | --- | --- |
| 1. | Do you any difficulties eating tough foods compared to 6 months ago? | +2 |  |
| 2. | Have you choked on your tea or soup recently? | +2 |  |
| 3. | Do you use dentures? | +2 |  |
| 4. | Do you often have a dry mouth? | +1 |  |
| 5. | Do you go out less frequently then you did last year? | +1 |  |
| 6. | Can you eat hard foods like squid jerky or pickled radish? |  | +1 |
| 7. | How many times do you brush your teeth in a day? (3 or more times/day) |  | +1 |
| 8. | Do you visit a dental clinic at least annually? |  | +1 |
| Total |  |  |  |

1. ***3Q/TMD questionnaire***

Q1. Do you have pain in your temple, face, jaw, or jaw joint once a week or more?

Q2. Do you have pain once a week or more when you open your mouth or chew?

Q3. Does your jaw lock or become stuck once a week or more?

1. ***Fonseca questionnaire***

**Foncesa questionnaire** consists of 10 questions answered as yes, no, sometimes. Yes answer is 10 points, sometimes answer is 5 points and no answer is 0 points. Those with a total score of 0-15 do not have TMJD, those with a score of 20-40 have mild TMD, those with a score of 45-60 have moderate TMJD, those between 70-100 points have severe TMD.

| Questions | NO | Sometimes | Yes |
| --- | --- | --- | --- |
| 1- Is it hard for you to open your mouth? |  |  |  |
| 2- Is it hard for you to move your mandible from side to side? |  |  |  |
| 3- Do you get tired /muscular pain while chewing? |  |  |  |
| 4 - Do you have frequent headaches? |  |  |  |
| 5- Do you have pain on the nape or stiff neck? |  |  |  |
| 6- Do you have earaches or pain in craniomandibular joints? |  |  |  |
| 7- Have you noticed any TMJ clicking while chewing or when you open your mouth? |  |  |  |
| 8- Do you clench or grind your teeth ? |  |  |  |
| 9- Do your feel your teeth do not articulate well? |  |  |  |
| 10- Do you consider yourself a tense (nervous) person? |  |  |  |

1. ***DC-TMD (Dentist)***

**
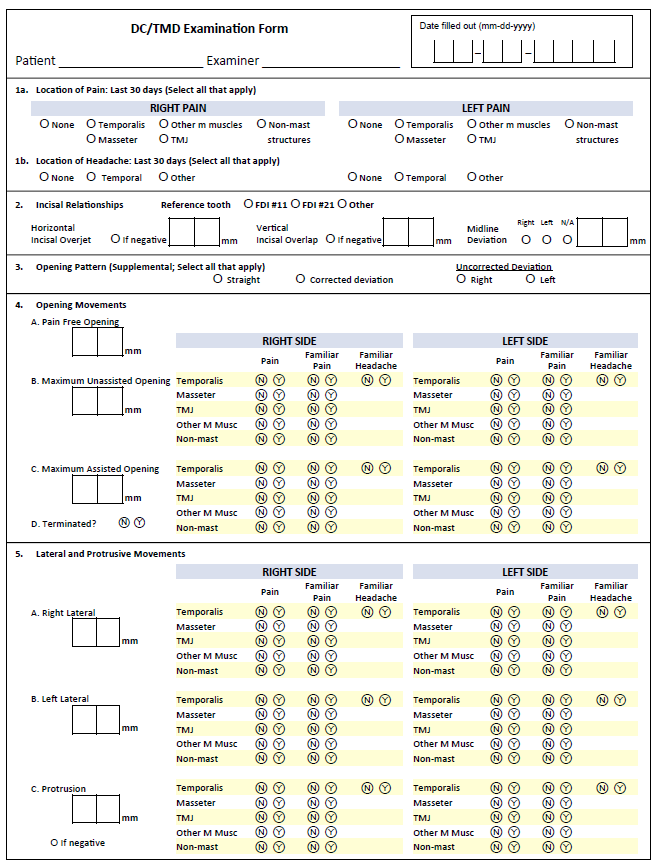
**

**
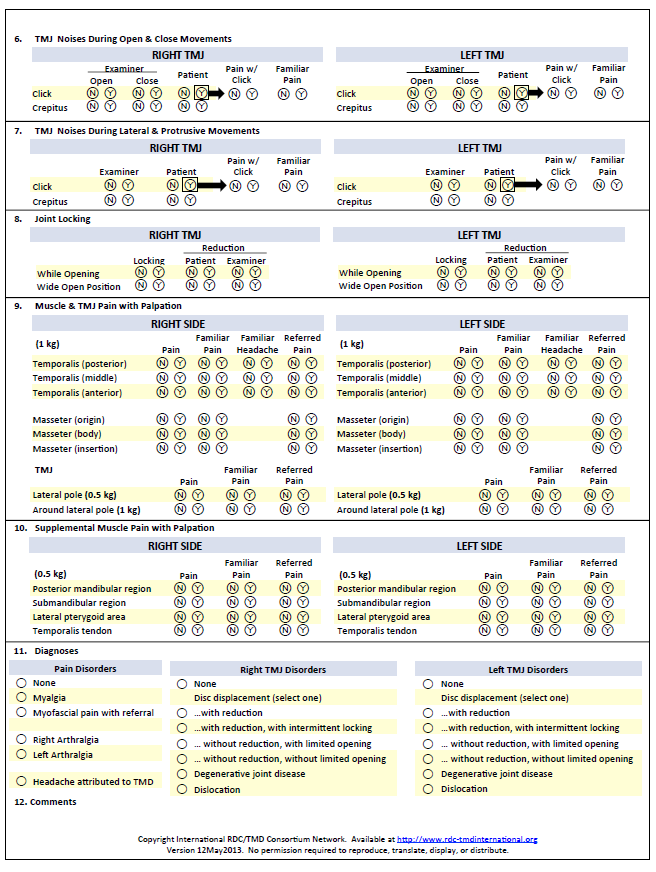
**

1. ***Mini nutritional assessment (MNA):***

***
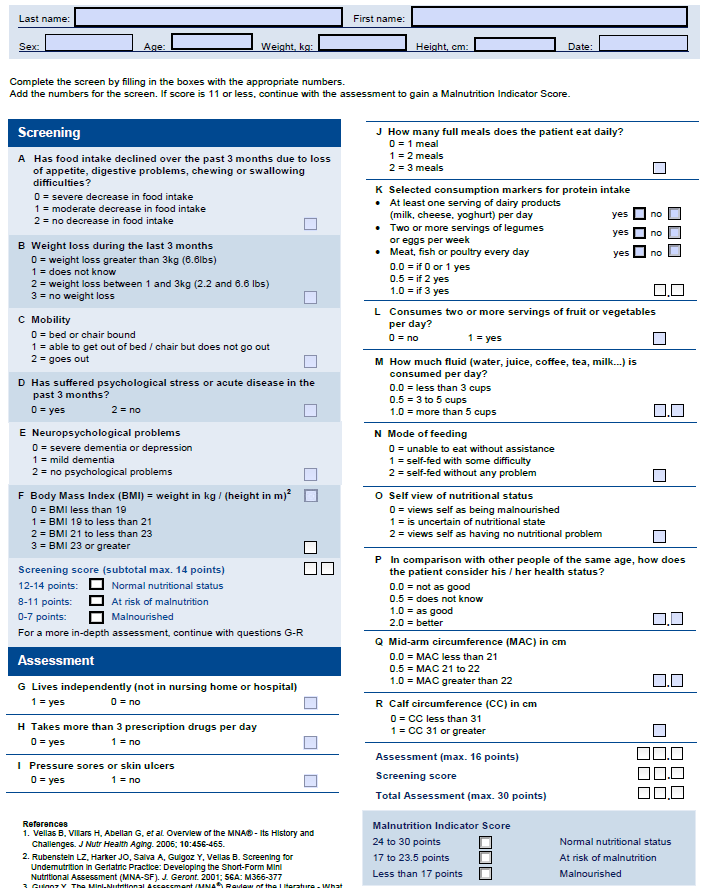
***

**Step 3: Microbiological examination**

Patients with **poorest oral hygiene** in screening and clinical assessment will go for third step i.e., microbiological sampling. Microbiological samples are collected at week 1 and week 4 (follow-up).

**Material required for each patient:**

1. Two 15 ml tube with 0.9% PBSc buffer.
2. One salivette tube with cotton roll inside.
3. Tongue scrapper.
4. One sterile cotton swap.
5. ***Sampling of saliva:***

The patients are disable and cannot deliver a traditional saliva sample. Therefore, saliva is sampled using a **sterile cotton roll which is embedded in a sterile tube (Salivette)**. The sterile cotton role will be placed using a forceps in the back of the oral cavity between the **lower back molar and the tongue** with the aim of collecting a saliva reservoir. The cotton roll is kept at the site for 30 seconds. The cotton role is transferred back to Salivette tubes and the lid is tightly closed. The samples are stored at -20 °C before being transported to the lab. The samples are processed, and microbial DNA is purified.

1. ***Sampling of tongue-coating:***

Samples from the dorsum of the tongue are collected using a tongue scraper (approximately 0.2 g). Tongue-coating is sampled by scraping the tongue two times. The coating is picked by the help of **sterile cotton swab** and transferred into a 15 ml tube containing 0.9 % PBS buffer. The samples are stored at -20 °C until transported to the lab for processing and microbial DNA purification.

1. ***Whole-mouth sampling*:**

A sample containing microorganisms from the whole cavity is sampled using a **sterile cotton swap**. The cotton swap is moved two times, on both side of inside the cheek, under the tongue, on the tongue and over teeth surface around the gingival margin. The tip of cotton swap is transferred to a 15 ml tube containing 0.9 % PBS buffer. The wooden part of the cotton swap is broken off. The samples are stored at -20 °C before being transported to the lab. The samples are processed, and microbial DNA is purified.

**Microbiological analysis**

The relative abundance between species of the tongue microbiome is determined by a 16S rRNA gene amplicon sequencing pipeline using Multiplex Illumina Sequencing at the Department of Biomedicine, Aarhus University. Additionally, the saliva and tongue samples will be analyzed by qPCR to determine the bacterial load.

**Step 4: FINAL OUTCOME SCORES (BASED ON SCREENING AND CLINICAL ASSESSMENT)**

1. **Oral health**


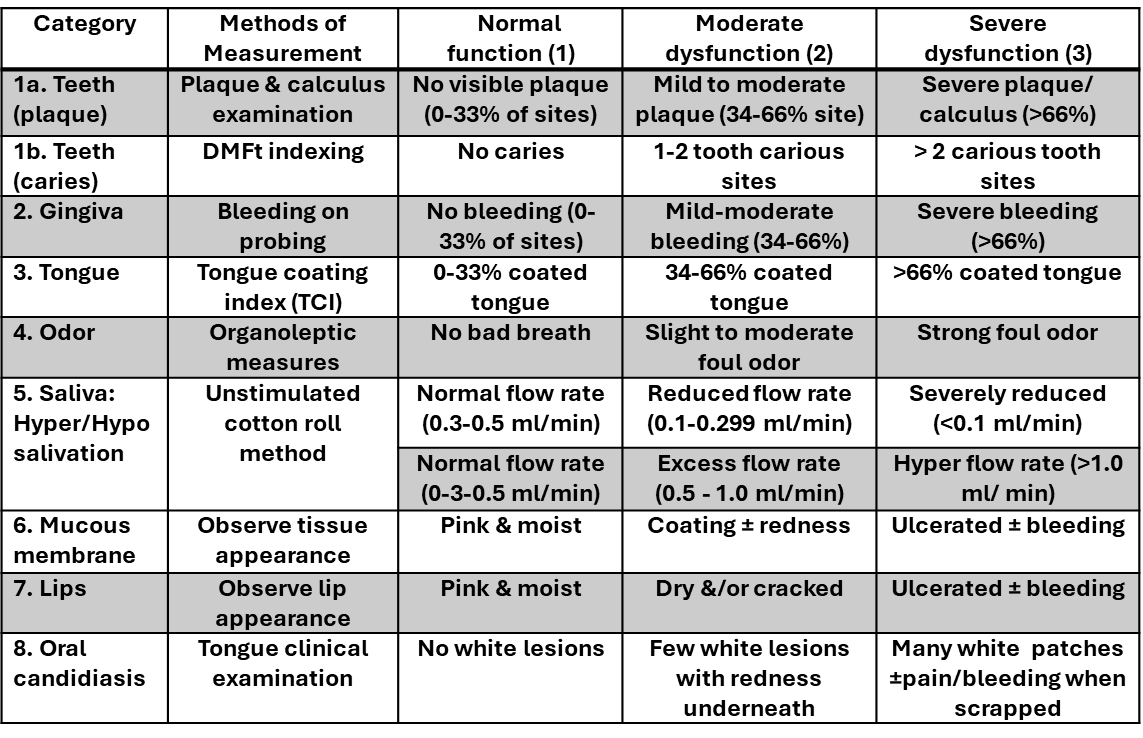


1. **Orofacial Health (Associated risk factors to oral care)**


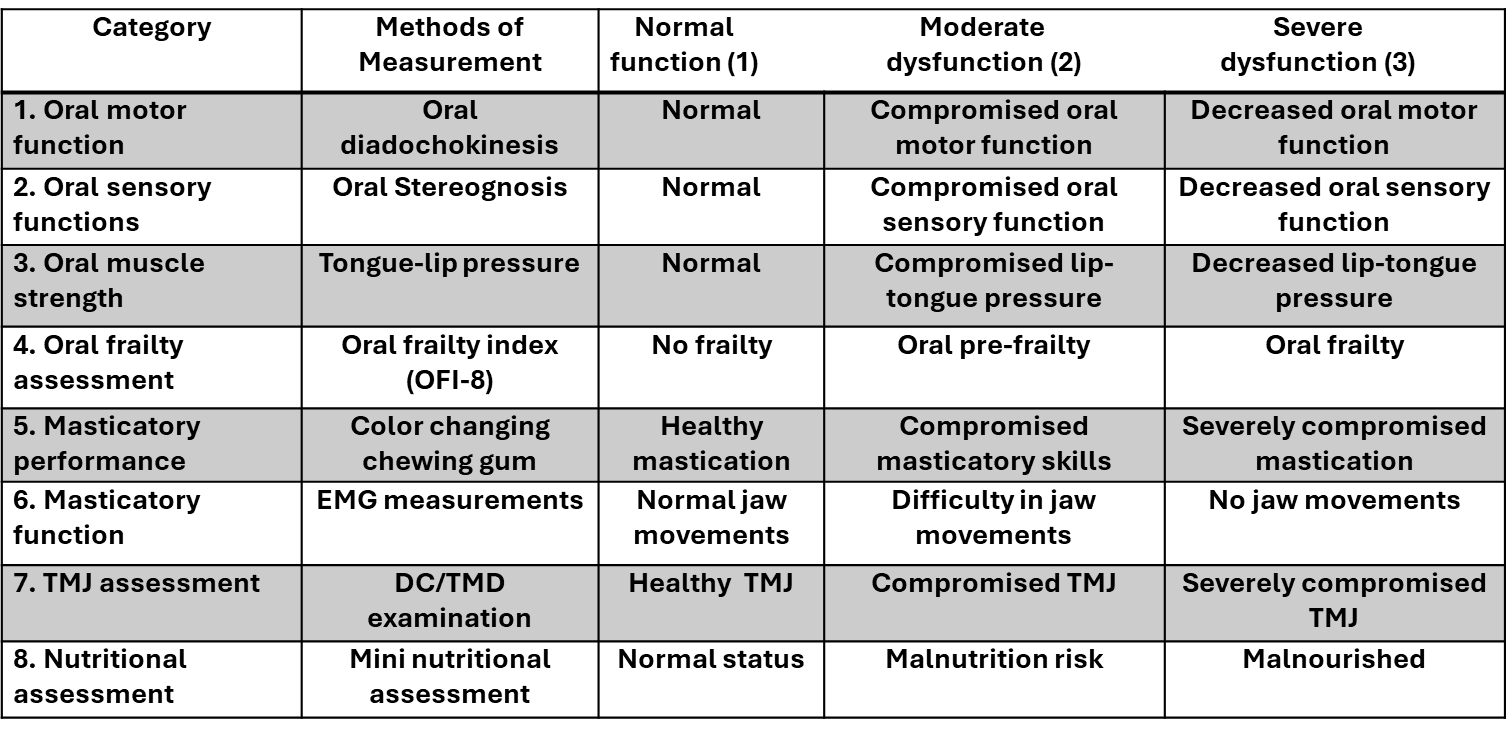


**Intervention for three weeks**

**Group 1: Existing Oral Care Plan**

**Wards S1, S3 and S6**

**OR**

**Group 2: MOHIT (new Rx plan)**

**Wards S2, S10 and S11**

**STEP 5A: EOCP PLAN AT HOSPITAL (HNRC)**

| **Individuals at HNRC** | **Standard oral care**  **(recommended clinical guidelines)** | **Supplemental oral care**  **(case-dependent)** |
| --- | --- | --- |
| **Patients with self-oral care** | **Instruction to brush twice daily, preferably after each meal Free to have any toothbrush they bring from home (small head/ big head/ electric soft bristle toothbrush) with fluoride toothpaste (1450 ppm)** | **Chlorhexidine mouth wash (0.12%)**  **Oral mucosal care Dental floss once a day Lip moisturizer for dry or cracked lips** |
| **Patients with swallowing/ eating/ motor/ cognitive difficulties (oral care by caregivers)** | **Cleaning of mouth for food debris and secretions before and after each intake of food and drinks**  **Use of small head soft bristle toothbrush and fluoridated non-foaming toothpaste (1450 ppm)**  **Tooth brushing in circulatory motion starting bucally, palatally and then to occlusal table twice a day after meal** | **Chlorhexidine mouth wash (0.12%)**  **Oral mucosal care Lip moisturizer for dry or cracked lips** |

**STEP 5A: MOHIT PLAN BASED ON FINAL OUTCOME SCORES (SCREENING AND CLINICAL ASSESSMENT)**

| **Category/ Items** | **Individualized oral care plan**  **Either after Breakfast/ Lunch/ Dinner** | **Normal function (1)** | **Moderate dysfunction (2)** | **Severe dysfunction**  **(3)** |
| --- | --- | --- | --- | --- |
| **1a. Teeth with plaque (Patients with self-care)** | **a) Pediatric toothbrush for 2 min**  **b) Interdental brush (if only patient eats by mouth)** | **XX*** |  |  |
|  | **a) Electric sonic toothbrush for 2 min**  **b) Interdental brush (if only patient eats by mouth)** |  | **XX** | **XXX** |
| **1a. Teeth (Patients dependent on caregivers)** | **a) Electric suction toothbrush 2 min**  **b) Interdental brush (if only patients eat by mouth)** | **XX** | **XX** | **XXX** |
| **1b. Teeth with caries** | **Fluoridated paste (>1000 ppm)** | **XX** | **XX** | **XXX** |
| **2. Tongue** | **Tongue scrapper after tooth brushing (back to front)** | **X** | **XX** | **XXX** |
| **3a/ 4a/ 8a. Gingiva/ Odor/ Candidiasis (Patients with self-care)** | **20 ml, 0.2% Chlorhexidine (CHX) mouth wash for 30 sec. in mouth, gargle & then spit ( after tongue scraping)** | **-** | **X** | **XX** |
| **3b/ 4b/ 8b. Gingiva/ Odor/ Candidiasis (Patients dependent on caregivers)** | **Dip toothbrush into 20 ml, 0.2 % CHX mouth wash and apply in the entire mouth covering tongue, tooth, gum for 30 sec. (after tongue scraping)** | **-** | **X** | **XX** |
| **5/6. Dry mucosa / Dry mouth** | **Oral moisturizer/ toothpaste with moisturizer** | **-** | **XX** | **XXX** |
| **7. Lips** | **Water-based moisturizer for lips** | **X** | **XX** | **XXX** |
| **9. Respiratory pathogens** | **Consult physician for probable pneumonia** | **-** | **-** | **-** |

X: one time/day

**If in any case the assessment cannot be completed, patients will be excluded.**

**Reason for exclusion: Cognitive/ Fatigue/ Mouth opening/ Other**

***Examiner/s sign and date:***

1. ***Dentist***
2. ***Nurses***
